# Small extracellular vesicle cargo as biomarkers in autoimmune rheumatic diseases: a systematic review

**DOI:** 10.1007/s00296-025-05953-w

**Published:** 2025-09-01

**Authors:** Michail K. Chatzopoulos, George E. Fragoulis, Martina Samiotaki, Maria G. Tektonidou, Petros P. Sfikakis, Eleni-Kyriaki Vetsika

**Affiliations:** 1 First Department of Propaedeutic Internal Medicine, Joint Rheumatology Program, School of Medicine, National and Kapodistrian University of Athens, Athens, Greece; 2 Institute for Bio-innovation, Biomedical Sciences Research Center “Alexander Fleming”, Vari, 16672 Greece; 3 Centre of New Biotechnologies and Precision Medicine (CNBPM), School of Medicine, National and Kapodistrian University of Athens, Athens, Greece

**Keywords:** Rheumatic diseases, Extracellular vesicles, Rheumatoid arthritis, Systemic lupus erythematosus, Differential diagnosis, Prognosis

## Abstract

Increasing evidence has shown the role of small extracellular vesicles (sEVs) in autoimmune rheumatic diseases (ARDs). This systematic literature review aims to evaluate the role of sEVs as biomarkers in ARDs, focusing on their molecular cargo and their utility for disease diagnosis, monitoring, and treatment response. A systematic search was conducted in MEDLINE/PubMed and Scopus from inception until July 2025, using the search terms; [(small extracellular vesicles) or exosomes) and ((rheumatic disease) or (rheumatoid arthritis) or (psoriatic arthritis) or (axial spondylarthritis) or (ankylosing spondylitis) or (systemic lupus erythematosus) or (Sjögren’s syndrome) or scleroderma or (systemic sclerosis) or myositis or polymyositis)]. Eligible studies were those reporting on sEV isolation from patient samples and comparing it with healthy individuals or controls with non-inflammatory conditions. The initial search yielded 1593 results, and 46 studies met the inclusion criteria. Literature reviews revealed that miRNAs and long non-coding (lnc)RNAs isolated from sEVs might serve as potential biomarkers for disease activity and treatment response in ARDs, mainly in rheumatoid arthritis (RA) and systemic lupus erythematosus (SLE). In addition, sEV miRNA-21 and miRNA-146a have been often described in studies in SLE patients, indicating their potential role in differentiating SLE from healthy individuals. Although proteomic studies identified disease-specific proteins within sEVs, there is no consensus among the limited studies reporting sEV proteins. Although numerous studies have examined the role of sEVs in ARDs, there is a lack of consensus in the findings. Further research using well-standardized methodologies is needed to ensure reliable and reproducible results.

PROSPERO 2025 CRD420250646438. Available from https://www.crd.york.ac.uk/PROSPERO/view/CRD420250646438.

## Introduction

Autoimmune Rheumatic Diseases (ARDs) are characterized by dysregulated immune responses, chronic inflammation and a wide range of clinical symptoms and signs from several organs [1]. While the exact pathogenic mechanism of ARDs remains widely unclear, it is now understood that a variety of factors, such as genetic profile, environmental triggers, and hormonal factors, contribute to their initiation and perpetuation [2]. The overlapping clinical manifestations of these diseases often lead to diagnostic challenges, requiring more specific diagnostic tests.

Developing novel diagnostic tools is crucial, as some ARDs, such as psoriatic arthritis (PsA), lack specific laboratory tests and rely primarily on clinical and imaging evaluation for diagnosis [3]. The use of biomarkers is pivotal not only for diagnosing ARDs but also for enabling personalized treatment approaches. As novel biological therapies continue to emerge, the ability to predict treatment response through biomarker profiling is becoming increasingly important, paving the way for more targeted and effective treatment plans.

Accumulating evidence suggests the important role of small extracellular vesicles (sEVs) as critical mediators of intercellular communication and modulators of cellular responses [4]. sEVs are saucer-shaped structures produced by almost all human cell types, with a size between 30 and 200 nm. They consist of a lipid bilayer membrane and contain a cargo of macromolecules such as proteins, lipids, and nucleic acids [5]. The nomenclature of extracellular vesicles has long been a source of debate in literature. The term “exosome” has been widely used interchangeably with sEVs. According to the Minimal Information for Studies of Extracellular Vesicles (MISEV 2023), the term exosome implies the biogenetic process that leads to the creation of an extracellular vesicle, while the term sEV only refers to the size of the examined vesicle, making exosomes a subpopulation of sEVs [4, 5].

Concerning ARDs, emerging data accentuates the important role of sEVs not only as contributors to the pathogenesis but also as promising biomarkers and potential therapeutic targets for these diseases [6]. The molecular cargo of sEVs consists of lipids, proteins, and RNAs. Among these molecules, non-coding RNAs (ncRNA) have been mostly investigated as potential biomarkers for various diseases. These molecules’ short half-life and chemical instability create discrepancies in studies examining ncRNAs, especially miRNAs [7]. Exosomal miRNAs are promising biomarkers as they are encapsulated in a lipid bilayer membrane, providing protection and enabling more reliable analysis [8].

This review aims to evaluate the existing literature on the isolation of sEV from the biological fluids of patients with ARDs and the comparison of their cargo to that of healthy individuals and controls of non-inflammatory conditions.

## Search strategy

The protocol for this review was registered in PROSPERO (CRD420250646438). A literature search was conducted in MEDLINE/PubMed and Scopus from inception until 15/07/2025 using the key terms: ((*small extracellular vesicles*) or *exosomes*) and ((*rheumatic disease*) or (*rheumatoid arthritis*) or (*psoriatic arthritis*) or (*axial spondyloarthritis*) or (*ankylosing spondylitis*) or (*systemic lupus erythematosus*) or (*Sjögren’s syndrome*) or (*scleroderma*) or (*systemic sclerosis*) or (*myositis*) or (*polymyositis*) following the Preferred Reporting Items for Systematic Reviews and Meta-Analyses (PRISMA) guidelines [9]. References of relevant articles were also screened for additional eligible studies not identified in the initial search. The eligibility of studies was assessed independently by two investigators (MKC and E-KV). Disagreements were discussed and resolved after consensus with the other authors (GEF and PPS).

Inclusion criteria: studies on isolating sEVs and their cargo from biological fluid samples from patients with ARD, in comparison to healthy individuals or controls with noninflammatory conditions (e.g., osteoarthritis). Exclusion criteria: non-English studies and those using animal models or cell cultures. Studies not adequately describing the method of sEV isolation and characterization are excluded.

## Results of literature selection and data extraction

**Fig. 1:**
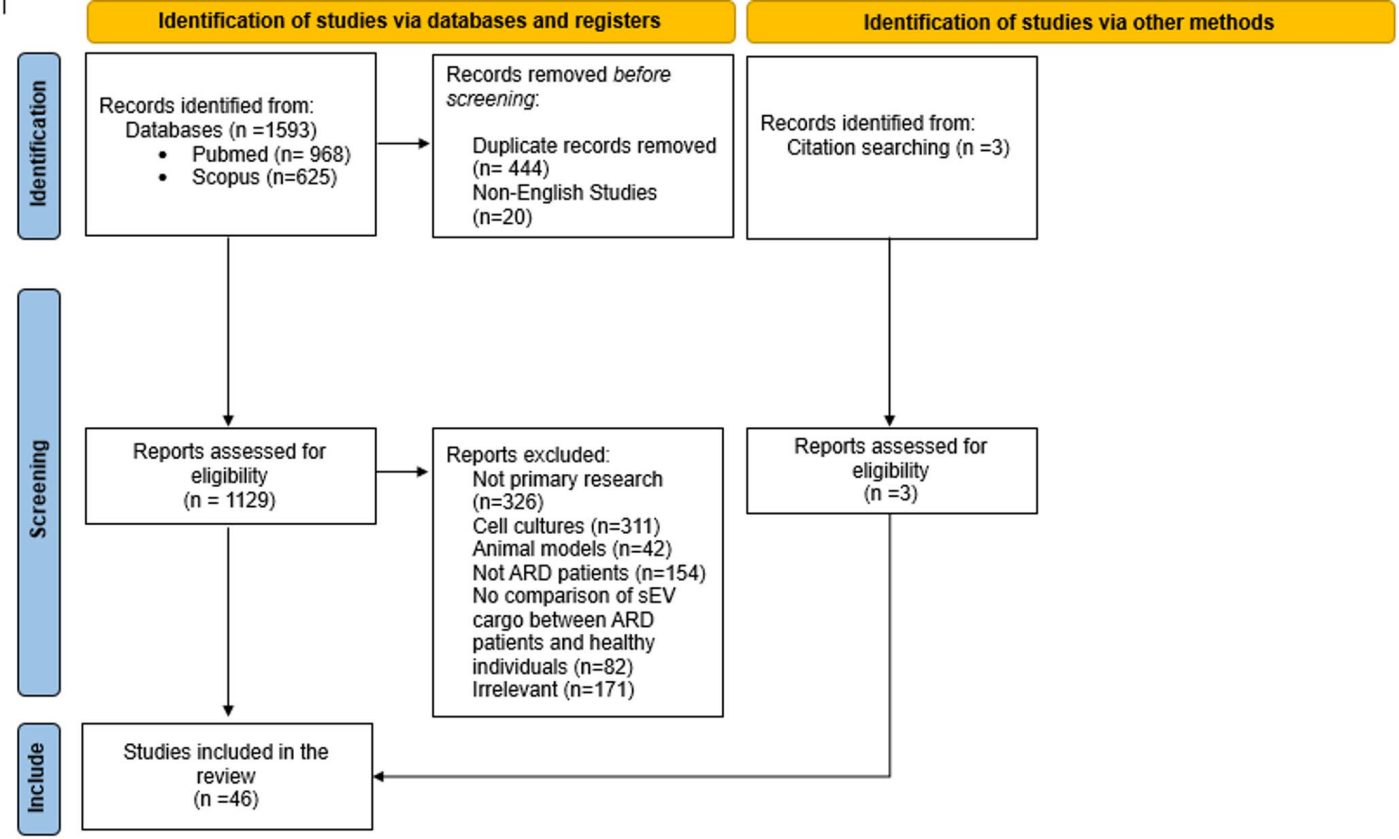
Flowchart

The initial literature search yielded 1593 publications (968 from PubMed and 625 from the SCOPUS database), of which 1129 were found to be non-duplicate records in English. Among these results, 803 consisted of primary research studies. A total of 154 studies were considered ineligible as they did not examine patients with ARDs. Another 311 publications were excluded because they reported on isolated sEVs from cell cultures, and 42 studies on isolated sEVs from animal models. Eighty-two studies did not compare the cargo of sEVs between healthy individuals and ARD patients, while 141 studies were deemed irrelevant to the topic of this review. Three additional studies were identified through the snowball approach by searching the references. Full texts of 46 studies were finally included in this review. A graphical representation of the above can be found in Fig. 1.

In the first section, we will summarize the bibliographic findings of studies comparing the sEVs cargo between patients with Rheumatoid Arthritis (RA) and controls. In this section, we will initially examine the ncRNA cargo of sEVs, followed by an analysis of their proteomic cargo. Next, we will present data from studies involving the exosomal ncRNAs and proteins in patients with Systemic Lupus Erythematosus (SLE). Finally, we will outline the data concerning the cargo of sEVs in patients with other ARDs.

## Rheumatoid arthritis (RA)

### Exosomal/sEV RNAs

To our knowledge, the first report of exosomal ncRNA isolation in patients with RA was published by Song et al. in 2014. In a cohort of 28 RA patients, they found that the sEVs long non-coding RNA (lncRNA) HOTAIR (HOX transcript antisense RNA) was significantly upregulated in serum compared to healthy individuals [10]. Recently, Wu et al. have shown a differential expression of sEV lncRNAs between patients with active RA and healthy controls, as well as among patient subgroups classified by their disease activity score 28 (DAS28), demonstrating that sEV lncRNA cargo analysis can provide valuable insights into disease activity [11]. Similarly, Shuai et al. showed a correlation between sEV lncRNAs RPS18P9 and SNGH31 with DAS28 [12], further supporting the potential of sEV lncRNAs as markers of disease activity.

Among exosomal ncRNAs, most research has focused on microRNAs (miRNAs). Various studies have documented differences in sEV miRNA expression between RA patients and healthy individuals. As shown by Lu et al., plasma-derived sEV miR-144-3p and miR-30b-5p were downregulated in RA patients, and these levels were inversely correlated with DAS28 score and anti-CCP antibody levels, indicating their potential as biomarkers for monitoring disease activity in RA [13].

Interestingly, in a large cohort of 231 RA patients and 185 healthy controls, another sEV miRNA molecule, miR-885-5p, could effectively distinguish between healthy controls and anti-CCP-negative RA patients, while miR-1268a appears to be expressed at lower levels, following methotrexate treatment initiation [14]. Moreover, clinical remission after methotrexate administration has also been correlated with sEV miRNA-1915-3p [15]. In the largest patient cohort identified in the present review, comprising 306 individuals with RA and an equal number of healthy controls, significant findings emerged regarding the expression of microRNAs in serum-derived sEVs. Specifically, miRNA-103a-3p, miRNA-10a-5p, miRNA-204-3p, miRNA-330-3p, and miRNA-19b were found to be significantly downregulated in the sEVs of patients compared to healthy controls [16]. These results highlight the potential role of circulating miRNAs within sEV to serve as diagnostic tools in RA, especially when assessed in well-powered studies [13]. Moreover, miR-204-5p and miR-204-3p, though different strands derived from the same precursor miRNA, are both reported to be downregulated in RA patients. Wu et al. observed reduced levels of miR-204-5p [17], while Hussain et al. reported a decreased expression of miR-204-3p [16]. This recurring pattern of downregulation within the miR-204 family may suggest its potential relevance as a biomarker in RA [13, 14]. Beyond ncRNAs, limited research has been conducted on sEV mRNAs in patients with chronic arthritis. Xue et al. demonstrated that the mRNA of the chemotactic molecule CCL5, which plays a significant role in the pathogenesis of inflammatory arthritis, is downregulated in the sEVs of RA patients [18]. A detailed summary of all relevant studies is provided in Table 1.

**Table 1:** Nucleic acid cargo of small extracellular vesicles in patients with rheumatoid arthritis

A/A	Study	Sample	No. of patients	No of comparators	sEV’s size (nm)	ncRNA	Major findings
1	Wu et al. [11]	Serum	13	5	N/A	TCONS_I2_00013502 ENST00000363624	lncRNA TCONS_I2_00013502 (elevated) and lncRNA ENST00000363624 (decreased), in RA patients compared to HC Potential biomarkers for RA diagnosis Combined with ACPA improved the accuracy of RA diagnosis
2	Lu et al. [13]	Plasma	24	24	50–150	miR-144-3p miR-30b-5b	Reduced levels in RA patients Negatively correlated with disease activity and ACPA levels Effectively distinguish RA patients from HC
3	Gong et al. [14]	Serum	231	185	90	miR-885-5p miR-6894-3p miR-1268a	Upregulated in RA patients Combined with ACPA, demonstrate high diagnostic accuracy for RA miR-885-5p can distinguish ACPA-negative RA patients from HC Decreased miR-1268a expression following methotrexate treatment, suggests its potential as a treatment marker
4	Shuai et al. [12]	Peripheral Blood	48	48	N/A	SNHG6, RPS18P9, RPL21P28, EBLN3P, FAM153CP, RPL23P8, SNHG31, NORAD, H3P6, DLEU2, TUG1, OIP5-AS1, CXXC4-AS1, OLMALINC, and NPHP3-AS1	12 lncRNAs (SNHG6, RPS18P9, RPL21P28, EBLN3P, FAM153CP, RPL23P8, SNHG31, NORAD, H3P6, DLEU2, TUG1, and OIP5-AS1) were upregulated, while three lncRNAs (CXXC4-AS1, OLMALINC, and NPHP3-AS1) were downregulated in RA patients. SNHG6 exhibited a negative correlation with CRP and ESR, while RPS18P9 and SNHG31 showed negative and positive effects on DAS28, respectively.
5	Xue et al. [18]	Serum	19	19	30–200	CCL5 mRNA NONHSAT193357.1	Decreased in RA patients compared to HC The expression level of lncRNA NONHSAT193357.1 was markedly reduced in RA patients compared to HC
6	Yang et al. [49]	Plasma	46	38	N/A	let-7a-5p, let-7b-5p, let-7d-5p, let-7f-5p, let-7 g-5p, let-7i-5p, miR-128-3p, miR-25-3p	Increased levels of let-7a-5p, let-7b-5p, let-7d-5p, let-7f-5p, let-7 g-5p, let-7i-5p, miR-128-3p, and miR-25-3p in RA patients let-7a-5p and miR-25-3p levels were associated with a rheumatoid factor-positive phenotype in RA patients
7	Hussain et al. [16]	Serum	306	306	30–150	miRNA-103a-3p miRNA-10a-5p miRNA-204-3p miRNA-330-3p miRNA-19b	Downregulated in RA patients compared to HC.
8	Yu et al. [50]	Peripheral Blood	13	10	N/A	has-miR-335-5 hsa-miR-486-5p	Elevated expression in RA compared to HC
9	Wu et al. [17]	Plasma	100	104	50–200	miR-204-5p	Downregulated in RA patients
10	Rodríguez-Muguruza et al. [51]	Peripheral Blood	28	28	30–150	miR-144-3p miR-451a miR-25-3p miR-15a-5p miR-107	Upregulated in RA patients
11	Lim et al. [15]	Serum	20 RA non remission	22 RA in remission	NA	hsa-miR-1915-3p	Negatively associated with the disease activity of RA
12	Wang et al. [52]	Plasma	25	N/A	89.5	miR-17	Increased and inversely correlates with Treg frequency in RA patients
13	Song et al. [10]	Plasma	28	5	N/A	HOTAIR	Upregulated in RA patients with RA

*sEV*, small extracellular vesicles; *No*, number; *ncRNA*, non-coding RNA; *RA*, rheumatoid arthritis; *HC*, healthy control; *miRNA*, micro-RNA; *lncRNA*, long-noncoding RNA; *ACPA*, anti-citrullinated protein antibodies; *CRP*, C-reactive proteins; *ESR*, erythrocyte sedimentation rate; *DAS*, Disease Activity Score; *N/A*, not available

### Exosomal/sEV proteins

In 2006, Skriner et al. were the first to conduct a proteomic analysis in synovial fluid-derived sEVs from RA patients, discovering that citrullinated proteins are present in these vesicles, contributing to the pathogenesis of the disease [7]. A more recent study by Alghamdi et al. in 2025 confirmed the presence of citrullinated proteins in the serum of RA patients [19]. Further research focused on the common protein identification markers of sEVs CD9, CD63, and CD81 tetraspanins in RA. Rydland et al. examined the proteomic profile of tetraspanin molecules on sEVs from patients with RA and healthy individuals, observing a distinct pattern of tetraspanin expression in the sEVs of RA patients (Table 2). They also found that responders to methotrexate had a unique high relative proportion of CD81 single-positive sEV compared to non-responders, suggesting tetraspanin expression in sEVs as a potential marker of response to methotrexate treatment [20].

**Table 2:** Proteomic cargo of small extracellular vesicles in patients with inflammatory arthritis

A/A	Study	Disease	Sample	No. of patients	No. of comparators	sEV’s size (nm)	Proteins	Major findings
1	Brenis Gómez et al. [25]	RA	Serum	9	8 OA 5 HC	119.4-219.9	45 differentially expressed sEV proteins	Higher levels of Annexin A2 (ANXA2), filaggrin (FLG), and fatty acid-binding protein 5 (FABP5) in OA sEV Higher levels of alpha-2-macroglobulin (A2M), apolipoprotein B (APOB), and fibronectin (FN1) in RA sEV
2	Alghamdi et al. [19]	RA	Serum	116	35	30–153	Citrullinated fibrinogen	Patients sEVs were more enriched in protein Half of the sEV samples that tested positive for anticitrullinated protein autoantibodies contained citrullinated fibrinogen (cFBG), a protein that plays a significant role in the development of rheumatoid arthritis (RA)
3	Rydland et al. [20]	RA	Plasma	8	5	50–200	CD9 CD63 CD81	CD9- or CD81-positive sEVs were more abundant in RA patients compared to HC, but double-positive sEVs were scarcer MTX-responders exhibited a distinctive pattern of tetraspanin expression
4	Huang et al. [21]	RA axSpA	Synovial Fluid	9 RA 9 axSpA	9 OA	60–120	1,678 detected	39 sEV proteins were highly expressed in axSpA, while 28 proteins were highly expressed in RA Lysozyme C levels in RA and axSpA were higher than in OA Sec24C was highly expressed in axSpA compared to other groups High PZP levels in RA compared to other groups. Complement and coagulation cascade proteins were upregulated in RA synovial sEV.
5	Qin et al. [22]	RA	Plasma	6	6	N/A	278 detected	32 upregulated and 5 downregulated proteins in RA patients compared to HC Among upregulated proteins, TTR, AGT, CD14, LBP, SAP/APCS, TNC, and COMP interact and are involved in inflammatory pathways related to RA
6	Tsuno et al. [23]	RA	Serum	33	10	50–100	TLR3	Upregulated in active RA patients
7	Yoo et al. [24]	RA	Serum	30 active	30 inactive	NA	AA LYVE-1	AA concentrations were higher in patients with high disease activity LYVE-1 concentration was lower in patients with higher disease activity
8	Skriner et al. [7]	RA	Synovial Fluid	5 RA	5 OA	30–200	Citrullinated proteins	Synovial sEV contained citrullinated proteins which act as autoantigens in RA

*No*, number; *RA*, rheumatoid arthritis; *axSpA*, axial spondyloarthritis; *OA*, osteoarthritis; *HC*, healthy controls; *sEV*, small extracellular vesicles; *MTX*, methotrexate; *SF*, synovial fluid; *PZP*, Pregnancy zone protein; *TTR*, transthyretin; *AGT*, angiotensinogen; *LBP*, lipopolysaccharide-binding protein; *SAP/APCS*, serum amyloid P-component; *TNC*, tenascin; *COMP*, cartilage oligomeric matrix protein; *TLR3*, Toll-like receptor 3; *AA*, amyloid A; *LYVE-1*; lymphatic vessel endothelial hyaluronan receptor 1; *N/A*, not available

Proteomic profiling of synovial fluid-derived sEVs from RA, axial spondyloarthritis (axSpA) and osteoarthritis (OA; comparators) patients revealed that sEV proteins could distinguish inflammatory from non-inflammatory arthritis. Additionally, the study showed that sEV proteins, such as Sec24C and pregnancy zone protein (PZP), could be used for diagnosing SpA and RA, respectively [21]. Another group of researchers confirmed the distinct proteomic cargo in plasma sEV of RA patients, discovering that proteins closely related to inflammatory or autoimmune diseases were upregulated in RA plasma-derived sEV [22]. Tsuno et al., analyzed the protein cargo of serum-derived sEV from RA patients and showed that the expression of some exosomal proteins, such as Toll-like receptor 3 (TLR3), can distinguish patients with active from those with inactive disease [23]. Additionally, other proteins, such as serum amyloid A (AA) and lymphatic vessel endothelial hyaluronan receptor 1 (LYVE-1) within sEV, also appear to be promising biomarkers as they significantly correlate well with disease activity in patients with RA [24]. In a more recent study, Brenis Gómez et al. compared the sEV protein cargo between OA and RA patients, finding distinct differences [25]. A detailed summary of the aforementioned studies is provided in Table 2.

### Exosomal/sEV ncRNAs and proteins in patients with systemic lupus erythematosus (SLE)

A substantial body of research on sEV cargo in patients with ARDs focuses on SLE, particularly in the discovery of ncRNAs as biomarkers for assessing disease activity. Ji et al. reported that the exosomal miR-122-5p levels in SLE patients were strongly correlated with disease activity (Systemic Lupus Erythematosus Disease Activity Index (SLEDAI) score) [26]. In a cohort of 20 SLE patients, two exosomal lncRNA molecules, the LINC00667 and DANCR, have been correlated with the SLEDAI score [27]. Similarly, Somparn et al. found an inverse correlation between the exosomal miR-146a concentration and SLEDAI score in patients with Juvenile Proliferative Lupus Nephritis (JPLN) [28]. miR-146a has also been investigated as a potential biomarker by Li et al., who reported its downregulation in exosomes of SLE patients, a finding consistent with the results of Dong et al. [29, 30]. The same molecule, miR-146a, has been highlighted by Perez-Hernandez et al. as an exosomal marker in urine, with a potential role in assessing the severity and the activity of Lupus Nephritis (LN) [31, 32]. Additionally, Tan et al. focused on the sEV miRNA miR-451a, another promising molecule that appears to correlate with disease activity and renal damage in SLE inversely [33].

sEV tRNA-derived small non-coding RNAs (tsRNAs) have emerged as a promising biomarker for diagnosing SLE and distinguishing SLE patients with and without LN. In one of the largest cohorts identified, including 253 SLE patients, tRF-Thr-TGT-4-M3 and tRF-Tyr-GTA-1-M2 were highly expressed in patients with LN compared to those without, while tRF-iMet-CAT-1, tRF-Ala-AGC-2-M4, and tRF-Tyr-GTA-1-M2 were highly expressed in patients without LN compared to healthy controls [34]. The same group of researchers previously demonstrated that tsRNAs were found to be upregulated in urine-derived sEVs of SLE patients with LN compared to those without LN and healthy controls, suggesting their potential role as urine biomarkers in LN [35].

As highlighted in the aforementioned studies, the urgent need for non-invasive methods to diagnose LN has driven research toward identifying molecular biomarkers for liquid biopsies. For instance, hsa-miR-4796-5p and hsa-miR-7974 have been proposed as potential biomarkers for diagnosing LN [36]. Similarly, Li et al. highlighted miR-21 and miR-155 as sEV biomarkers for the same purpose [29]. In addition, miR-31, miR-107, and miR-135b-5p have been reported as potential urine exosomal biomarkers for the prediction of treatment response in patients with LN [37]. A panel of three sEV miRNAs in urine, including miR-21, miR-150, and miR-29c, was found by Solé et al. to predict disease progression in LN patients [38]. Detailed results are summarized in Table 3. Although non-coding RNAs in the sEV of patients with SLE have been extensively studied, there is a paucity of data regarding sEV proteins in these patients. Therefore, this represents a promising area for future research.

**Table 3:** ncRNA and proteins in small extracellular vesicles of patients with systemic lupus erythematosus

A/A	Study	Disease	Sample	No. of patients	No. of comparators	sEV’s size (nm)	ncRNA	Major findings
1	Peng et al. [27]	SLE	Plasma	20	20	100	LINC00667 DANCR	Upregulated in the patients Positively correlated with disease activity
2	Ji et al. [26]	SLE	Plasma	43	19	107	miR-151a-3p miR-1180-3p miR-1246 miR-122-5p	miR-151a-3p was lower in SLE patients, while miR-1180-3p, miR-1246, miR-122-5p were higher miR-122-5p was correlated with SLEDAI activity, positively correlated with dsDNA level, while negatively correlated with C3 and C4 level
3	Yang et al. [34]	SLE	Serum	253	80	NA	tRF-iMet-CAT-1 tRF-Ala-AGC-2-M4 tRF-Tyr-GTA-1-M2 tRF-Thr-TGT-4-M3 tRFTyr-GTA-1-M2	tRF-iMet-CAT-1, tRF-Ala-AGC-2-M4 and tRF-Tyr-GTA-1-M2 showed high expression in SLE group without LN than in HC tRF-Thr-TGT-4-M3 and tRFTyr-GTA-1-M2 were higher expressed in LN group than in SLE without LN
4	Somparn et al. [28]	SLE (Juvenile Proliferative LN)	Plasma	12	NA	100–150	miR-146a	Upregulated following 12 months of treatment and inversely correlated with lupus disease severity, anti-double-stranded DNA antibody concentration and proteinuria (urine protein–creatinine ratio)
5	Chen et al. [36]	SLE	Serum	116 SLE-LN	116 SLE	127	hsa-miR-4796-5p hsa-miR-7974	Upregulated in LN patients compared to SLE patients
6	Flores-Chova et al. [53]	SLE	Plasma	96	25	NA	miR-101-3p miR-16-5p LINC01986 AC087257 AC022596.1 LINC01015	Upregulated in LN patients
7	Chen et al. [35]	SLE	Urine	173	24	129	tRF3-Ile-AAT-1 tiRNA5-Lys-CTT-1	Increased in the urine of patients with LN compared to SLE patients without LN and HC
8	Song et al. [54]	SLE	Plasma	10	20	30–200	Various miRNAs and proteins	407 upregulated and 596 downregulated miRNAs 84 upregulated (CRP, LGALS3BP, IGHG1, C1QB, C4BPA, etc.) and 13 downregulated (IGHV3OR15-7, HPR and HYDIN) proteins
9	Tan et al. [33]	SLE	Serum	42	21	30–110	miR-451a miR-16 miR-451a	miR-451a and miR-16 were downregulated miR-451a expression correlated with disease activity and renal damage
10	Li et al. [29]	SLE	Serum	38	18	NA	miR-21 miR-155 miR-146a miR-155	miR-21 and miR-155 were elevated miR-146a levels were decreased Higher levels of miR-21 and miR-155 in LN patients compared to non-LN SL? patients
11	Peres Hernandez et al. [32]	SLE	Urine	41	20	70–170	miR-146a	Increased sEV miR-146a levels compared to urine microvesicles in SLE patients, especially in patients with LN Association with disease activity
12	Garcia-vives et al. 2020 [37]	SLE	Urine	22 LN Responders	21 LN non-responders	93.4 ± 36.6	miR-31 miR-107 miR-135b-5p MiR-135b	Responders expressed increased levels of sEV miR-31, miR-107, and miR-135b-5p in urine and renal tissue compared to non-responders miR-135b exhibited the best predictive value to discriminate responders
13	Dong et al. 2019 [30]	SLE	Serum	10	10	100	miR-146a	Downregulated in SLE patients
14	Sole et al. [38]	SLE	Urine	45	20	85.5 ± 33.4	miR-21 miR-150 miR-29c	Detected early renal fibrosis and predict disease progression in LN patients
15	Tangtanatakul et al. [48]	SLE	Urine	13 SLE-LN	18 SLE inactive-LN	40–200	Let-7a miR-21	Downregulated in active LN patients
16	Peres Hernandez et al. [31]	SLE	Urine	38	12	21–140	miR-146a miR-335 miR-302d miR-200c	Increased levels in active LN patients. sEVmiR-146a indicates the presence of active LN miR-302d elevated in inactive LN
17	Sole et al. [55]	SLE	Urine	32	35	65.35 ± 34.7	miR-29c	Correlated with the degree of renal chronicity but not with the creatinine levels

*SLE*, Systemic Lupus Erythematosus; *sEV*, small extracellular vesicles; *LN*, Lupus Nephritis; *HC*, healthy controls; *SLEDI*, Systemic Lupus Erythematosus Disease Activity Index; *dsDNA*, double stranded; *NA*, not available

### Sjögren’s syndrome (SS), polymyositis/dermatomyositis (PM/DM), systemic sclerosis (SSc)

Few studies have explored the differences in sEVs in patients with other ARDs, beyond RA and SLE, compared to healthy controls. We mention these studies for the completeness of the current review.

Regarding Sjögren’s Syndrome (SS), researchers have identified sEV miRNAs in mouth rinse-derived sEV as potential diagnostic biomarkers [39]. In a cohort of 18 patients, Peng et al. discovered that ferroptosis-related proteins, such as ceruloplasmin and transferrin, are downregulated in plasma-derived sEV of SS patients compared to healthy individuals. Apart from the discovery of a distinct pattern of protein expression in sEV of patients with SS, the results of this study highlight that ferroptosis may play a role in the pathogenesis of SS [40].

In 2021, Li et al. examined the RNA profile in plasma sEV of patients with Dermatomyositis (DM) and identified three differently expressed miRNAs and three lncRNAs participating in autophagy, which might serve as clinical biomarkers [41]. Zhong et al. suggested that the sEV miRNA profile in patients with DM can distinguish patients from HC as well as patients with different clinical profiles of the disease [42]. Proteomic analysis performed by Meng and colleagues revealed a unique sEV protein signature in Polymyositis/Dermatomyositis (PM/DM) patients, including proteins of the complement system and the coagulation cascade, suggesting their pathogenic role [43]. Finally, Wermuth et al. reported differences in the levels of profibrotic and antifibrotic miRNAs in sEVs of limited and diffuse Systemic Sclerosis (SSc) patients and healthy individuals [44]. Detailed results are summarized in Table 4.

**Table 4:** Cargo of small extracellular vesicles in patients with Sjögren’s syndrome, Systemic Sclerosis, Polymyositis, and Dermomyositis

A/A	Study	Disease	Sample	No. of patients	No. of comparators	sEV’s size (nm)	Proteins/ncRNAs	Major findings
1	Guiot et al. [56]	SSc	Plasma	91	43	90–200	miR-584-5p, miR-744-5p, miR-1307-3p and miR-10b-5p	These four sEV miRNAs can serve as a biomarker to differentiate between SSc patients with and without ILD
2	Peng et al. [40]	SS	Plasma	18	18	131.8	Ferroptosis related proteins	24 differentiated expressed proteins in pSS compared to HC, 17 of which were upregulated proteins, while seven were downregulated and some of these promote ferroptosis
3	Yamashiro et al. [39]	SS	Mouth rinse	24	24	145	let-7b-5p miR-1290	In combination, can be used for diagnosing SS.
4	Meng et al. [43]	PM/DM	Plasma	24	9	116.4	Various proteins (coagulation and complement proteins)	50 and 34 differentially expressed in DM and PM patients, respectively 16 proteins were common to DM and PM patients Complement components C1QB and C1QC were significantly upregulated in DM/PM patients compared to HC Coagulation-associated proteins (FGA, FGB, FGC, and VWF) were significantly higher in DM/PM patients compared to HC
5	Li et al. [41]	DM	Plasma	32	22	120	hsa-miR-1246 hsa-miR-125a-3p hsa-miR3614-5p ENST00000584157.1 ENST00000523380.1 ENST00000560054.1	44 upregulated and 9 downregulated miRNAs in DM patients compared with HC hsa-miR-1246, hsa-miR-125a-3p and hsa-miR3614-5p were decreased in DM patients after treatment. 313 upregulated lncRNAs and 139 downregulated lncRNAs in DM patients compared with HC The 3 miRNAs and the 3 lncRNAs formed a network and participated in autophagy.
6	Zhong et al. [42]	DM	Plasma	10	5	122	Various miRNAs	38 miRNAs were significantly upregulated in sEV from patients with DM-ILD-MDA5 Ab^+^ compared to HC 21 miRNAs were significantly downregulated 73 sEV miRNAs were differentially expressed between DM-nonILD-MSA16^-^ and HC
7	Wermuth et al. [44]	SSc	Serum	6	12	75–200	Various miRNAs	Increased expression of let-7 g, miR17, miR23b and miR29a (profibrotic) in diffuse SSc patients Decreased expression of let-7a, miR125b, miR140, and miR146a (antifibrotic) in diffuse SSc patients Differential antifibrotic miRNA profile between diffuse SSc patients and limited SSc patients

*sEV*, small extracellular vesicles; *SS*, Sjögren’s Syndrome; *SSc*, Systemic Sclerosis; *PM*, polymyositis; *DM*, dermomyositis; *HC*, healthy controls; *ILD*, interstitial lung disease

## Discussion

Our literature review revealed an imbalance in the number of studies between ncRNA and proteomics analysis on sEVs in ARDs, with the greatest emphasis on nc RNAs. Among ARDs, RA and SLE are the most extensively investigated in this context, yet studies on sEV proteins remain relatively low. In this review, a key observation is the increasing use of non-invasive methods for sEV isolation, such as saliva from SS patients and urine from LN patients. These non-invasive approaches, by allowing repeated non-invasive sampling, have the potential to provide valuable insights not only for diagnosis but also for prognosis and treatment response in these diseases.

Since their discovery, sEVs have been attributed to numerous physiological and pathological roles. Their structure and biological composition make them ideal messengers for cell-to-cell communication and efficient carriers of information, traveling through biological fluids and transferring molecules away from their cellular origin. A growing body of knowledge implicates these structures in the pathogenesis of various diseases, and increasing research focuses on identifying sEV molecules that can serve as biomarkers or liquid biopsies, providing new, efficient methods for diagnosing and tracking disease progression. While new techniques for accurately identifying different types of sEVs have been proposed, questions remain regarding the optimal strategies for their isolation and characterization [45].

Efforts to use the cargo of sEVs to distinguish ARD patients from healthy individuals have consistently shown promising results. In this review, we aimed to compile studies comparing the sEV cargo between patients and healthy individuals. Reviewing the current bibliography, we compiled 46 scientific papers reporting on the cargo of sEV in patients with ARDs, with most research focused on miRNAs contained within sEVs. These small ncRNAs can alter gene expression and have been extensively investigated as potential biomarkers or therapeutic targets in ARDs [46]. In this review, we analyzed thirty-three studies that highlighted sEV miRNAs as promising biomarkers to diagnose ARDs or assess the activity and the response to treatment. Despite the relatively large number of studies, there seemed to be no universal consensus on the miRNA molecules that differ in various ARDs and healthy individuals.

Among all the exosomal miRNAs examined, miR-146a emerged as a promising biomarker in SLE in multiple studies [28, 29, 32]. miR-146a has been proposed as a biomarker in various ARDs in the past [47]. Additionally, based on findings from multiple studies, sEV miR-21 appears to play a complex and context-dependent role in SLE and LN. Elevated levels of miR-21 have been reported in the serum of SLE with high SLEDAI scores and in LN patients, suggesting a potential association with disease activity and renal involvement. Conversely, urinary sEV studies indicate that miR-21 may be downregulated in patients with active LN, while also serving as a biomarker for early renal fibrosis and disease progression. These contrasting patterns highlight the importance of biological source and disease stage in interpreting miR-21’s diagnostic and prognostic value [29, 38, 48].

Beyond RNA cargo, proteomic analysis of sEVs has provided compelling evidence of their involvement in ARDs. Several studies have identified disease-specific protein signatures in sEVs, with implications for the pathogenesis and diagnosis of these diseases. For example, citrullinated proteins within sEVs in rheumatoid arthritis patients suggest a direct role in propagating autoimmunity [7]. Additionally, proteomic analyses of sEVs in SS and PM/DM revealed key differences in immune-related and ferroptosis-associated proteins, further emphasizing their relevance in disease pathology [40, 43].

It is important to note that although there are only a limited number of studies concerning the proteomic profile in sEVs in the studied populations, discrepancies in methodology exist. Different techniques, which are not always well-standardized, continue to be employed. To address this issue, it is essential to adopt standardized methods that can precisely detect proteins. Techniques such as Liquid Chromatography Mass Spectrometry (LC-MS) are highly recommended, as they allow for the detection of a wide array of proteins with great accuracy and reproducibility. This approach would ensure more reliable and consistent results, facilitating the identification of protein biomarkers in small extracellular vesicles across autoimmune rheumatic diseases.

Another important point from the reviewed literature is the significant heterogeneity in the number of participants across studies. Some studies include a substantial number of patients and comparators, which strengthens the findings. However, some studies include a very small number of patients, which represents a limitation. This variability in sample sizes should be considered when interpreting the results.

We acknowledge certain limitations in the present review. As mentioned before, the studies included exhibit heterogeneity both in terms of study design and sample size. Despite these limitations, we believe our review is comprehensive, encompassing a broad range of articles from the existing literature, both older and more recent, and highlighting the current knowledge gap regarding the molecular content of small extracellular vesicles. To our knowledge, this is the first review to systematically summarize findings on both miRNAs and proteins contained within these vesicles in the context of autoimmune rheumatic diseases.

We believe that our systematic review offers readers of a rheumatology journal a roadmap for a new biomarker in rheumatic diseases, which is currently under investigation. It is noteworthy that our review focuses on small extracellular vesicles rather than extracellular vesicles in general, and it summarizes the existing knowledge while highlighting the gaps for future research.

## Conclusion

sEVs appear to be promising biomarkers for autoimmune rheumatic diseases. While non-coding RNAs, such as miR-146a, miR-21, and miR-204, demonstrate potential for diagnosing and monitoring ARDs, variability in study methodologies highlights the need for standardized techniques. Proteomic profiles also hold diagnostic promise but require further validation. Non-invasive sampling methods are particularly promising for improving patient care. Further research with larger, standardized cohorts is essential to translate these findings into clinical practice and fully realize the potential of sEVs.

## Declarations

### AI declaration

AI was not used in manuscript preparation; no editing services were used.
